# Microplastic Contamination and Ecological Status of Freshwater Ecosystems: A Case Study in Two Northern Portuguese Rivers

**DOI:** 10.3390/ijerph192315956

**Published:** 2022-11-30

**Authors:** Andreia Ribeiro, Carlos Gravato, João Cardoso, Carlos Alexandre Ribeiro, Maria Natividade Vieira, Carolina Rodrigues

**Affiliations:** 1 Landscape Laboratory, Rua da Ponte Romana, Creixomil, 4835-095 Guimarães, Portugal; 2 Faculty of Sciences of University of Lisbon & Centro de Estudos de Ambiente e Mar (CESAM), University of Lisbon, Campo Grande, 1749-016 Lisbon, Portugal; 3 Department of Biology, Campus de Gualtar, University of Minho, 4710-057 Braga, Portugal; 4 Department of Biology, Faculty of Sciences of the University of Porto, Rua do Campo Alegre, s/n, 4169-007 Porto, Portugal

**Keywords:** benthic macroinvertebrates, ecological status, freshwater, microplastic, sediments, aquatic organisms

## Abstract

Background: Most European rivers have not yet achieved “good” ecological status. In addition, the presence and abundance of microplastics (MPs) in freshwater is a matter of great concern to the scientific community. Methodology: This study assesses the ecological status of four sampling sites of Selho (S1–S4) and Costa-Couros (C1–C4) rivers (Guimarães, Portugal), and the abundance of MPs in sediments and benthic macroinvertebrates (Oligochaeta) from those sites. Results: All sites of both rivers under higher pressure did not reach a “good” ecological status (S2–S3, C2–C4) based on the macroinvertebrate community. High levels of nutrients were observed at all sites in both rivers (except C2), especially phosphorus. In the Oligochaeta’s gut of Costa-Couros river, the high number of MPs seems to be associated with their weight (95.25 ± 25.61 to 1069.00 ± 385.30 MPs g/fresh weight), suggesting the existence of malnutrition and digestive disorders, whereas the highest number of MPs in gut (134.00 ± 30.36 to 558.6 ± 100.70 MPs g/fresh weight) were found for the heaviest organisms of Selho. Conclusions: Thus, sites with higher ecological status do not necessarily have lower abundance of MPs. In the sediments, urbanization seems to be the main driver for MP contamination. MP contamination is pervasive across the sediments and Oligochaeta’s gut in both rivers. Since MPs have the potential to cause harm to environmental and human health, it is essential to monitor not only the ecological status of freshwaters, but also emerging pollutants such as MPs.

## 1. Introduction

Rivers have always played a crucial role in the development of societies, providing several services of great importance, from drinking water to energy production and transportation [1]. However, they are considered one of the most threatened ecosystems in Europe [2,3]. With the implementation in 2000 of the Water Framework Directive (WFD; Directive 2000/60/EC [4]), the definition of water quality according to its uses was replaced by the creation of a uniform system that allows the adaptation of general quality objectives to the specific environmental conditions for each type of water body. This gave rise to the concept of “ecological status” in assessing aquatic ecosystems. However, 18 years after its implementation, 53.5% of European rivers still failed to achieve at least a “good” ecological status (or potential), and 4.6% still presented an unknown ecological status [5].

Hydromorphological and diffuse pollution are the major pressures affecting European rivers, representing almost 70% of all pressures, 34% and 33%, respectively [5]. The physical modifications caused by hydrological (e.g., low flows, water abstraction, and flash floods) and morphological stress (e.g., straightening, bank fixation, and removal of riparian vegetation) affect the natural functioning of the riverine ecosystems, reducing the connectivity and habitat quality, and influencing the aquatic communities [6]. Additionally, diffuse or non-point sources indirectly discharge a great variety of pollutants, often chemicals or microorganisms, into the “receiving water bodies, via runoff and subsurface flow to surface waters” [7], resulting in great difficulty in controlling its sources. Furthermore, diffuse sources can cause microplastic (MP, i.e., plastic fragment less than 5 mm in length) pollution [7]. MPs are emerging persistent pollutants that have quickly been recognized as a threat due to their ubiquity and potential to impair ecosystem functions and services, organisms, and human health [8,9]. They are categorized into primary and secondary based on their sources. Primary MPs are intentionally manufactured on a microscale (e.g., pellets, personal care products) or result from the abrasion of large plastic objects during manufacturing, use, or maintenance (e.g., tire erosion during driving or abrasion of synthetic textiles during washing [10]). Most primary MPs come from uncontrolled human activities, the main entry routes being road runoff (66%) followed by treated effluent from wastewater treatment plants (25% [10]). Secondary MPs can result from the fragmentation of large plastics items improperly discarded in the environment by mechanical abrasion, biological degradation, or photodegradation [11,12,13]. Achieving a microsize, these small plastic fragments are easily transported by wind and currents, reaching even the most remote areas [13,14]. Once in the natural environments, they can be ingested by aquatic organisms having the potential for bioaccumulation and causing adverse effects in ecosystems [15,16,17]. Furthermore, once ingested, they can also transfer dangerous organic chemicals into the food chain [18].

Rivers are one of the major pathways of MP residues from land-based sources to oceans [19,20], and estimates suggest that more than 60 billion MP particles enter the ocean from rivers every day [21]. Even though more than 96% of the research studies on MP pollution were performed in the marine environment [22], recently, studies have focused on freshwater systems [23,24,25,26,27] and showed that the abundance of MPs in freshwater environments could be higher than in the marine one (e.g., [24]). Moreover, in both freshwater and marine environments, the sediments have always shown significantly higher levels of MPs than the water column [28].

Sediments provide habitat, substrate, and food to various organisms. An example of sediment-dwelling organisms is benthic macroinvertebrates, which are well known for being a major food resource for fish and one of the most important constituents of fluvial ecosystems [29]. Recent studies suggest that the ingestion of small MPs (<0.3 mm) by freshwater benthic macroinvertebrates can cause malnutrition, digestive disorders, and possibly affect larval development and emergence of imagoes in dipteran species [30,31]. Klein et al. [32] also demonstrated that ingestion of MPs by freshwater Oligochaeta (*Lumbriculus variegatus*) causes a reduction in their survival and body weight, although MPs mixed in the sediment affect Oligochaeta more than MPs that are layered on the sediment surface. Furthermore, contaminants accumulated in macroinvertebrates are likely to be transferred throughout the aquatic food web, with ensuing toxicological effects [33]. Therefore, contaminated sediment poses a great threat to the health of the entire aquatic ecosystem [34].

According to previous studies carried out in European rivers, urbanization is a major driver for the deterioration of the ecological status [35] and for MP contamination in freshwater ecosystems (e.g., [25,26,36]). Therefore, we tested the hypothesis that sites with greater urban influence show lower ecological status and a higher abundance of MPs. In order to address this hypothesis, we assessed (1) the ecological status of two riverine systems included in the municipality of Guimarães (the Selho and Costa-Couros rivers), at sites with different land uses, and (2) the presence and abundance of MPs in the sediments and in the gut of benthic macroinvertebrates collected in the same rivers and sites.

## 2. Materials and Methods

### 2.1. Study Area Description and Sampling Sites

**Figure 1:**
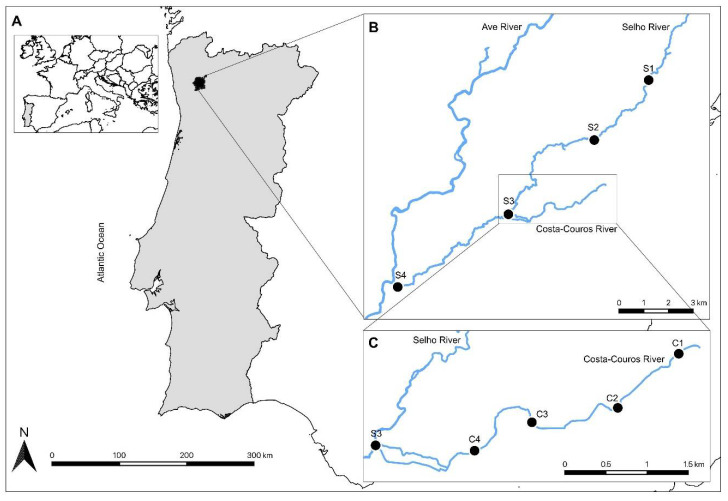
Geographical situation. (**A**) Location of the study area (Guimarães municipality; filled in black) in Portugal mainland. Location of the sampling sites of the (**B**) Selho river (S1 to S4) and (**C**) Costa-Couros river (C1 to C4).

This study was conducted in two urban Northern Portuguese rivers integrated in Guimarães municipality (Figure 1A): the Selho and the Costa-Couros. The Selho river (Figure 1B) is almost entirely integrated (>95%) within the municipality of Guimarães [37]. It has its spring in Santa Marinha, in Fafe municipality (spring altitude: 580 m) [37,38] and runs for approximately 21 km through urbanized, agricultural, and industrial areas, before flowing into the Ave river in the parish of Gondar, in Guimarães municipality [38,39]. The Costa-Couros river (Figure 1C) is fully integrated within the Guimarães municipality. It springs from Penha Mountain (spring altitude: 550 m) and runs for about 6.2 km through nine heavily urbanized parishes before flowing into the Selho river in Veiga de Creixomil [40].

Four sampling sites were selected in the Selho (S1 to S4; Figure 1B) and Costa-Couros (C1 to C4; Figure 1C) rivers to assess the ecological status and to evaluate the presence and abundance of microplastics (MPs). These sites were selected to include different land uses (Table 1). The Selho river sites are located in an agricultural and forestry area (S1), an urban area (S2), and agricultural areas (S3 and S4) of Guimarães municipality. The Costa-Couros river sites are situated in four main areas of the city of Guimarães, which are crossed by this river, namely, the City Park, the most extensive recreational area of the city (C1), the Parque das Hortas and the City Market, both situated in urban areas (C2 and C3, respectively), and in Veiga de Creixomil (C4), a peri-urban agricultural area.

### 2.2. Ecological Status Assessment

Benthic macroinvertebrates were monitored in the early summer of 2020, following the national guidelines for the Water Framework Directive (WFD) implementation [41]. Briefly, benthic macroinvertebrates were collected in all existing habitats using a hand net (mesh-size of 500 µm) and fixed in situ with ethanol 90% (*v*/*v*). In the laboratory, the organisms were sorted out, counted, and identified up to a family level (except for Oligochaeta—to subclass level) [42]. The ecological status was assessed by determining the North Invertebrate Portuguese Index (IPtI_N_ [43,44]), and the final quality value was expressed as the Ecological Quality Ratio (EQR). The EQRs were obtained by dividing the IPtI_N_ value by the reference value for each specific river type, with the Selho and Costa-Couros rivers belonging to the “small-sized streams of North of Portugal” type. An ecological quality class was also assigned (I—“high” to V—“bad”) [43].

Water physicochemical parameters were seasonally monitored (summer and autumn of 2020; winter and spring of 2021). Water temperature, pH, dissolved oxygen (concentration and percentage saturation), conductivity, salinity, and total dissolved solids were measured in situ using a portable multiparameter prove (HI9828; Hanna Instruments). Water samples were collected (±5 cm from the surface) at all sites and transported to the laboratory for further analysis. Nutrients (nitrates, nitrites, ammonium ion and total phosphorus) and chemical oxygen demand were determined using multiparameter bench photometers (HI83200 and HI83214; Hanna Instruments). Biochemical oxygen demand was determined by measuring the amount of oxygen in the water sample after 5 days of incubation at 20 °C (±1 °C) in the dark. The obtained results were compared with the thresholds established for the “good” ecological status in Northern Portuguese rivers [43].

### 2.3. Microplastics Quantification

Sediment and biological samples were collected to evaluate the presence and abundance of microplastics in the Selho and Costa-Couros rivers during the summer of 2020. For the sediment sampling, a transept was traced in a deposition zone, with the samples being collected ±3 cm from the top layer (total area of 30 cm × 30 cm). A total of five replicates per site, with 1 m between them, were collected and placed in properly labeled glass jars (1 L). For the biological samples, 15 replicates per site of benthic macroinvertebrates (Oligochaeta, Lumbricid) were collected using a hand net and preserved in 70% ethanol.

The extraction and quantification of MPs in sediment and biological samples followed Masura et al. [45] and Prata et al. [46,47], with slight modifications. The sediment samples were dried (60 °C; ±72 h), sieved (5 mm, 1 mm, and 0.5 mm), and weighed. The MPs present in the sediments with granulometry greater than 0.5 mm were separated and quantified using the density separation technique. The quantification of MPs between 0.5 and 1 mm was performed using a binocular magnifying glass (Leica EZ4 HD). In case of doubts, a needle test was performed (i.e., heating the tip of a thin needle and poking a suspected particle under the stereomicroscope [48]).

The extraction of the MPs from the sediment samples with particle size lower than 0.5 mm was performed by density separation (NaCl 5M; 1:3 ratio), mixing the sediment with a saturated NaCl solution, shaken vigorously for 3 min, and left to set down during the night. The supernatants containing MPs were vacuum-filtered onto glass fiber membranes (Watman, Grade GF/F: 0.7 μm; diameter: 47 mm), treated to remove the organic matter (30% H_2_O_2_ + FeO_4_S_7_H_2_O), and stained with Nile Red dye (1 μg/mL of ethanol). In the biological samples, organisms were weighed on a precision scale, digested (with 10% KOH; 60 °C, ±48 h), filtered, and stained with Nile Red.

The quantification of MPs lower than 0.5 mm in sediments and biological samples was performed after drying the filters (at room temperature in the dark) in glass Petri dishes. MPs were counted with an optical microscope (Leica DM300, Leica Microsystems: Heerbrugg, Switzerland) in a dark room, under 470 nm blue light (Optimax™ OFK-450A, Spectro-UV: New York, NY, USA). Using an orange filter, particles presenting red fluorescence (with defined edges) were counted as MPs.

### 2.4. Quality Assurance and Quality Control

The ubiquity of MPs requires additional caution during the experience to avoid contamination and data adulteration. Accordingly, precautionary measures were taken. Throughout sampling and the sample examination/process, the use of plastic material was avoided, preferably using glass or aluminum vials/equipment and stainless-steel utensils. Cotton lab coats were used, all the material was previously acid-washed before use, and samples were covered with aluminum foil when not used or processed. All solutions (NaCl, Nile Red, etc.) were previously filtrated before being used. All working spaces were thoroughly cleaned (alcohol). Procedural blanks were also applied [47].

### 2.5. Statistical Analysis

Spatial and seasonal variations of water physicochemical parameters in the Selho and Costa-Couros rivers were subjected to an ordinary two-way ANOVA. All datasets were subjected to the Shapiro–Wilk normality test to assess Gaussian distribution, selected for its adequacy for smaller sample sizes (N = 12) [49]. In the analysis of the physicochemical parameters, the ROUT method (Q = 0.1%) was applied to identify and remove definitive outliers.

The datasets containing the number of MPs in the sediments and organisms (Oligochaeta) collected from the Selho and Costa-Couros rivers were also subjected to the Shapiro–Wilk normality test to assess Gaussian distribution. When normal distribution was verified, an ordinary one-way ANOVA and Tukey’s multiple comparison test were performed. When it was not verified, nonparametric Kruskal–Wallis and Dunn’s multiple comparison tests were performed. In the analysis of the Oligochaeta weight used in the MP experiments, definitive outliers were identified and removed according to the ROUT method (Q = 0.1%). To assess the correlation between the number of MPs in the sediment and in Oligochaeta’s gut, linear regressions were fitted.

Comparisons between the number of MPs found in the two rivers (sediment and Oligochaeta’s gut) were made using an unpaired t-test if the normal distribution was verified. If data were not normally distributed, the nonparametric Mann–Whitney test was applied.

All statistical analyses were performed using GraphPad (GraphPad Prism version 9.0.0 (121) for Windows). A significance level of 0.05 was considered for all tests.

## 3. Results

### 3.1. Ecological Status

According to the IPtI_N_ index based on the benthic macroinvertebrate community, the Selho river presented “high” to “poor” ecological status (Table 2). The most upstream and downstream sites (S1 and S4, respectively), situated in forestry (S1) and agricultural areas (S1 and S4), were the only sites of this river with at least a “good” ecological status. The same index revealed that the ecological status of the Costa-Couros river decreased from the upstream to downstream sites, with C1, located in the City Park, being the only site of this river presenting a “good” ecological status (Table 2).

Most general physicochemical parameters analyzed in Selho and Costa-Couros rivers showed significant differences among sites and seasons (Table S1). In both rivers, the parameters dissolved oxygen (concentration and %saturation), pH, water temperature, and biochemical oxygen demand were more influenced by seasonal than spatial factors (Table S1). On the contrary, the parameters conductivity, total dissolved solids, salinity, nitrites, ammonium ion, and total phosphorus were more influenced by spatial than seasonal factors (Table S1). However, nitrates (NO_3_^−^) and chemical oxygen demand (COD) were not concordant between the two rivers. While in Costa-Couros river, NO_3_^−^ and COD varied more seasonally and spatially, respectively, the opposite occurred in Selho river (Table S1).

Overall, the Selho river presented a “good” ecological status considering all the physicochemical parameters analyzed, except for total phosphorus. High levels of phosphorus (*p* > 0.10 mg/L) were found at all sampling sites of Selho river, especially at S1, located in a forestry and agricultural area, when compared to the remaining sites (especially S3 and S4) (Table 3). The presence of phosphorus was more influenced by spatial than seasonal factors (spatial: 53.18%; F (3, 32) = 65.11; *p* < 0.0001; seasonal: 8.17%; F (3, 32) = 10.00; *p* < 0.0001; interaction: 29.95%; F (9, 32) = 12.22; *p* < 0.0001) (Table S1; Figure S1).

In Costa-Couros river, only C2, situated in an urban green area, presented a “good” ecological status considering all the physicochemical parameters analyzed. Phosphorus concentrations above the threshold value established for the “good” ecological status (*p* > 0.10 mg/L) were found at C1, C3, and C4 (Table 3). For this parameter, the spatial factor was stronger than the seasonal, but the interaction between them was the strongest factor (spatial: 38.86%; F (3, 32) = 166.6; *p* < 0.0001; seasonal: 4.15%; F (3, 32) = 17.80; *p* < 0.0001; interaction: 54.50%; F (9, 32) = 77.89; *p* < 0.0001) (Table S1; Figure S2). Concentrations of ammonium ion above the established threshold (NH_4_^+^ >1 mg/L) were observed in C4 during summer and autumn (Table 3). Furthermore, high levels of this parameter were observed in C3 during autumn. For ammonium ion, the spatial factor was the strongest, followed by the interaction of spatial and seasonal differences (spatial: 41.81%; F (3, 32) = 95,329; *p* < 0.0001; seasonal: 24.68%; F (3, 32) = 56,263; *p* < 0.0001; interaction: 33.51%; F (9, 32) = 25,471; *p* < 0.0001) (Table S1; Figure S2).

### 3.2. Analysis of Microplastics Contamination

**Figure 2:**
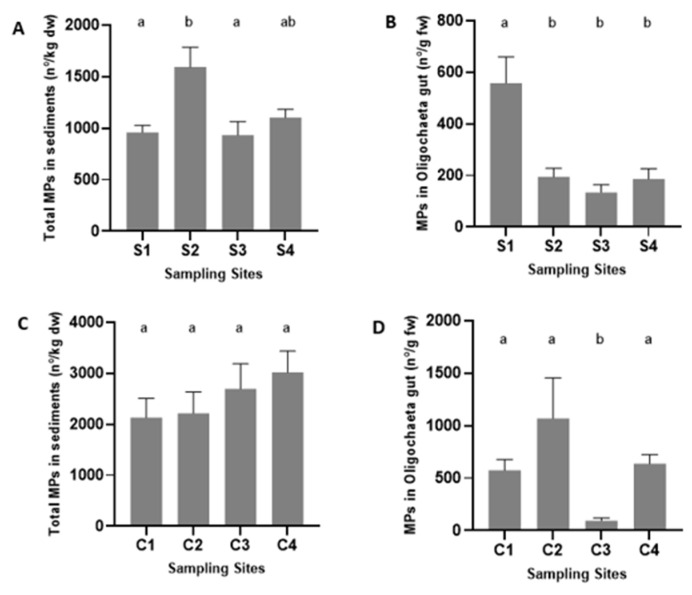
Total number of microplastics (MPs) found in Selho river ((**A**), sediments; (**B**), Oligochaeta’s gut) and Costa-Couros river ((**C**), sediments; (**D**), Oligochaeta’s gut). Data are presented as the mean ± SEM (sediment: *n*= 5; organisms: *n* = 15). Different letters indicate significant differences (*p* < 0.05).

**Figure 3:**
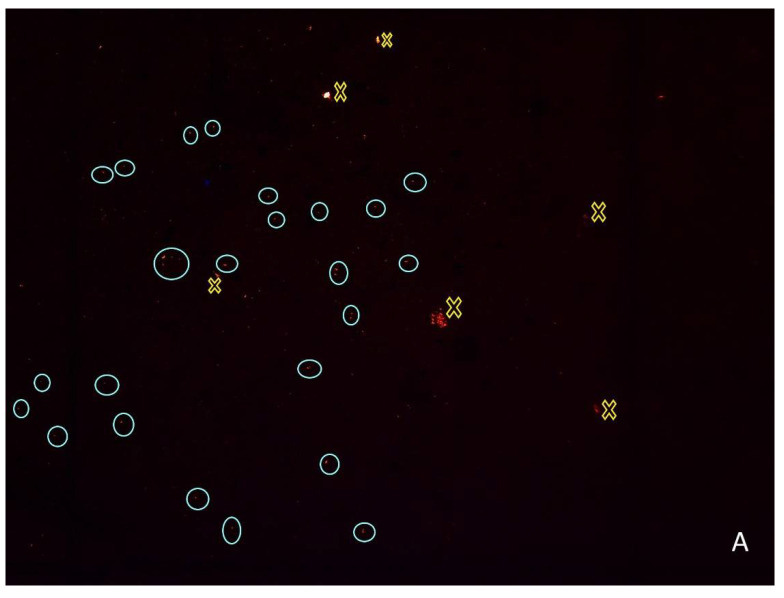

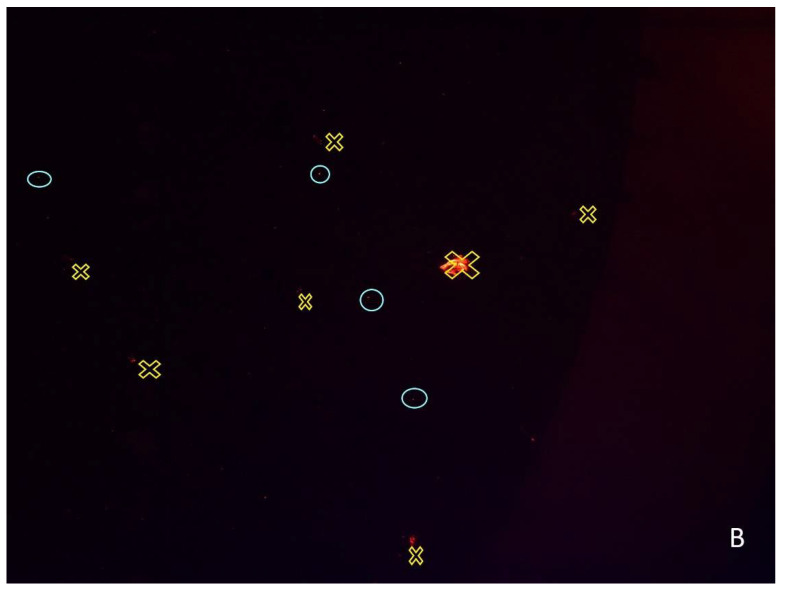
Random photos of a 100 mm^2^ area of a glass fiber membranes (in a total of approximately 1734.94 mm^2^, i.e., total area of the filter) containing MPs extracted from the Oligochaeta’s gut (**A**) and the sediment (**B**) of Costa-Couros river sampling sites. MP particles presented a red fluorescence according to Prata et al. [47]. Circles symbolize which particles were counted as MPs, while crosses symbolize those not counted as MP particles.

The results showed that MP contamination is widespread both in the sediment (Figure 2A,C) and in the digestive tract of Oligochaeta (Figure 2B,D) collected at all sampling sites of Selho and Costa-Couros rivers (Figure 3). Selho river showed an abundance of MPs between 932.5 ± 130.9 particles·kg^−1^ of dry weight and 1593.2 ± 190.3 particles·kg^−1^ of dry weight, and Costa-Couros river showed an abundance of MPs between 2130.3 ± 377.6 particles·kg^−1^ of dry weight and 3018.2 ± 416.8 particles·kg^−1^ of dry weight.

Significant differences were observed among Selho river sites for the number of MPs in the sediment (F (3, 16) = 5.845, *p* = 0.0068; Figure 2A), with a higher number of MPs in S2 (1593 ± 190.3 MPs·kg^−1^ of dry weight in S2), compared to the S1 (958.7 ± 69.26 MPs·kg^−1^ of dry weight) and S3 (932.5 ± 130.9 MPs·kg^−1^ of dry weight) sites. When MP sizes found in the sediments (>0.5 mm vs. <0.5 mm) were compared at each sampling site, there were more MPs with size <0.5 mm than MPs with size >0.5 mm at all sites (S1: t = 13.91, df = 4.002, *p* = 0.0002; S2: t = 8.381, df = 4.000, *p* = 0.0011; S3: t = 7.036 df = 4.002, *p* = 0.0021; S4: t = 13.60, df = 4.002, *p* = 0.0002). Significant differences were also observed among Selho river sites for the number of MPs existing inside the gut of Oligochaeta, with higher abundances occurring in S1 (558.6 ± 100.7 MPs·g^−1^ of fresh weight) compared to the remaining sites (H = 20.54, *p* = 0.0001; Figure 2B). The size (in µm) of the MPs ingested by Oligochaeta collected from Selho river ranged from 10 to 70 µm (Table 4), but the size of the ingested MPs did not differ significantly among sites. Comparing the weight of the Oligochaeta sampled at the sampling sites of Selho river, organisms from S1 and S4 had significantly higher weight compared to the organisms from S2 and S3 (F (3, 53) = 11.73; *p* < 0.0001; Table 5).

In Costa-Couros river, significant differences among sites were found for the number of MPs existing in the digestive tract of Oligochaeta, with a lower abundance of MPs occurring in C3 (95.25 ± 25.61 MPs·g^−1^ of fresh weight) compared to the remaining sampling sites (H = 23.23; *p* < 0.0001; Figure 2D). Results also showed that Oligochaeta ingested particles of different sizes ranging from 20 to 90 µm (Table 4), and the size of the MPs ingested by Oligochaeta was significantly different between C3 and C4 (F (3, 36) = 4.224; *p* = 0.0117). Significant differences among sites were also found for the weight of Oligochaeta, with higher weights occurring in C3 compared to the other sites (F (3, 46) = 36.88; *p* < 0.0001; Table 5). Although no significant differences were found among sites of Costa-Couros river for the number of MPs in the sediments (F (3, 16) = 0.9520, *p* = 0.4391; Figure 2C), there were significantly more MPs with size < 0.5 mm than MPs with size >0.5 at all sampling sites (C1: t = 5.631, df = 4.000, *p* = 0.0049; C2: t = 5.243, df = 4.000, *p* = 0.0063; C3: t = 5.469, df = 4.000, *p* = 0.0054; C4: t = 7.182, df = 4.000, *p* = 0.0020).

**Figure 4:**
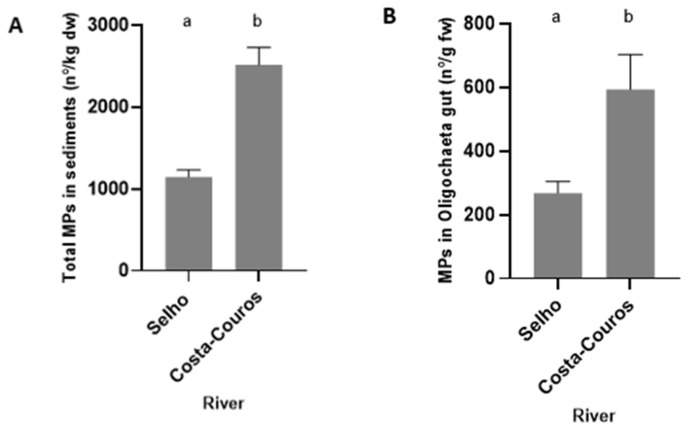
Total number of MPs extracted in the sediment (**A**) and Oligochaeta’s gut (**B**) of all sampling sites in the Selho and Costa-Couros rivers. Data are presented as the mean ± SEM (sediment: *n* = 20; organisms: *n* = 60). Different letters indicate significant differences (*p* < 0.05).

In both the Selho and the Costa-Couros rivers, no correlation was found between the numbers of MPs in the sediment and the number of MPs in Oligochaeta’s gut (Selho: R^2^ = 0.0351; F(1, 58) = 2.111; *p* = 0.1517; Costa-Couros: R^2^ = 0.0277; F(1, 58) = 1.651; *p* = 0.2039). When the two rivers were compared, significant differences were found for the abundance of MPs in the sediment (t = 5.967, df = 38; *p* < 0.0001) and inside the gut of Oligochaeta (U = 1245; *n*_1_ *= n*_2_ *=* 60 *p* = 0.0033), with Costa-Couros river presenting a greater abundance of MPs in both cases compared to Selho river (Figure 4).

## 4. Discussion

### 4.1. Ecological Status

The Water Framework Directive (WFD [4]) requires that the ecological status of a river is determined by the ecological quality element most affected by human activity (i.e., the element with the worst ecological status). In this sense, neither the Selho nor the Costa-Couros river reached a “good” ecological status because of the results obtained with the biological quality element analyzed (below “good” in S2 and S3 in Selho river and C2 to C4 in Costa-Couros river) and/or the presence of high levels of nutrients in both rivers (Table 3), especially phosphorus (exceeded maximum limit value established for the “good” ecological status at all sites, except in C2 in Costa-Couros river).

Regarding the physicochemical parameters, phosphorus is a fundamental element for plant growth and one of the key components of inorganic fertilizers [50,51]. This nutrient is not readily available, and plants do not use it efficiently, which leads to a continuous application in agricultural fields. Therefore, an uncontrolled accumulation of phosphorus in the soil [51] may later be leached into aquatic ecosystems by surface runoff or soil erosion [1,51] and trigger eutrophication processes, threatening aquatic life [1,50,51]. In Selho and Costa-Couros rivers, the presence of phosphorus seems to be more influenced by spatial than seasonal factors, as significant differences were found between sampling sites but not between seasons (Table S1). In the Selho river, S4 showed lower phosphorus concentration compared to the other sampling sites (S1 to S3), which may be due to the existence of more naturalized banks in S4 than the other sites. This may have resulted in a greater capacity of the riparian strip of this site to act as a “filter”, reducing the amount of nutrients and suspended sediments carried in runoff destined for the river [52,53]. In Costa-Couros river, higher phosphorus concentrations were observed in C3 (urban area) and C4 (peri-urban agricultural area) throughout the year. These two sites are located after the Costa-Couros river reappears at the surface after being under the city of Guimarães. This may suggest a continuous discharge of effluents when the river is under urban soil [54], reinforcing the influence of urban pressure in the river’s water quality.

The concentration of ammonium ion also exceeded the threshold value for the establishment of “good” ecological status at C4, with high concentrations of this nutrient in surface waters generally associated with contamination from raw sewage, industrial effluent, or fertilizer inflow [51,55]. Higher values of this nutrient in C4 (peri-urban agricultural zone) compared to C3 (urban zone) in summer and autumn seem to be associated with agricultural activities practiced on marginal land, since nutrients from fertilizers applied on agricultural fields may be transported to surface watercourses through irrigation channels, erosion, or rain water [51].

In both study rivers, the sites that did not reach at least a “good” ecological status based on the benthic macroinvertebrate community were those under higher urban pressure (S2, C2, and C3) or simultaneously under urban and agricultural pressure (S3 and C4), with increased hydromorphological modifications. These include the loss of riparian vegetation (C4) and the replacement of natural banks by artificial walls (S2, S3, C2, and C3), resulting in a decrease in riverbank vegetation and contributing to the reduction in infiltration in favor of surface runoff [37], and the presence of physical structures in the riverbed (S2 and C2), altering river dynamics, and acting as obstacles to water drainage and sediment transport [38]. Moreover, all sampling sites with ecological quality below “good” showed low diversity of organic (e.g., low abundance of macrophytes leading to a low value of families that depend on them as habitat and sources of food [42]) and inorganic (e.g., S2, S3, C2, and C3 there was a predominance of fine substrate, with this substrate often referred to as poor in terms of diversity [56]) habitats in the river bed. These factors have negatively impacted the macroinvertebrate communities in these sites, leading to a decline in the diversity of benthic macroinvertebrate species, a prevalence of taxa more tolerant to anthropogenic pressures (such as organisms of the phylum Mollusca, class Turbellaria, subclass Oligochaeta, and family Chironomidae).

The sites that achieved at least a “good” ecological status based on the macroinvertebrate community (C1, S1, and S4) showed fewer hydromorphological modifications than the other sites and presented more diverse habitats in the riverbed, providing better conditions for the development of a more stable and diverse community of benthic macroinvertebrates. As expected, in the Costa-Couros river, C1, which is located at the City Park, achieved the highest ecological status in this river (“good” ecological status). However, in Selho river, although S1 showed a “good” ecological status, the highest ecological status (i.e., “high” ecological status) was obtained in S4. The flow conditions in S4 led to the presence of a high number of organisms of the Hydropsychidae family [42], which was essential for the increase in the value of the IPtI_N_ index.

### 4.2. Microplastic Contamination

Microplastic (MP) contamination is pervasive across the sediments of the Selho and Costa-Couros rivers. The comparison of the abundance of MPs in the sediments of Selho and Costa-Couros rivers with other rivers is limited due to the lack of standardization of methodologies and measurement units, as well as because MPs’ abundance can be spatially and temporally variable [19]. Both rivers presented higher and similar abundances of MPs in the sediments compared to those reported in other European rivers. For example, they showed higher abundances of MPs than those reported for Tame (165 particles·kg^−1^ of dry weight [36]), Kelvin (161–432 particles·kg^−1^ of dry weight [19]), and Thames (66 particles 100 g—which is the equivalent to 660 particles per kilogram [23]) rivers in UK or Vistula river in Poland (190–580 particles·kg^−1^ of dry weight [57]). However, MPs in the sediments of Selho and Costa-Couros rivers were similar to those found in the sediments of Rhine (228 to 3763 particles·kg^−1^ of dry weight) and Main (786 to 1368 particles·kg^−1^ of dry weight) in Germany [58], and especially to those found in other Mediterranean rivers, such as the Ebro (2052 ± 746 particles·kg^−1^ of dry weight [59]) and the Henares (maximum of 2910 particles·kg^−1^ of dry weight [60]) rivers, both located in Spain. In Portugal, most studies evaluating the presence and abundance of MPs in the sediments were oriented toward marine ecosystems, with MPs being only analyzed in sediments of the Antuã (Aveiro) and Lis (Leiria) rivers. Our study revealed that Selho and Costa-Couros rivers have a higher abundance of MPs in the sediments than the Antuã river (18 to 629 particles·kg^−1^ of dry weight [25]) but very similar to those found in the Lis river (102.22 to 2206.59 particles ·kg^−1^ of dry weight [27]).

The spatial distribution of MPs in the sediments of both studied rivers showed different patterns. In Selho river, S2, located in an urbanized area, stood out for having more MPs in sediments compared to the remaining sites (agricultural and forestry areas). Similar results were also observed in previous studies conducted in rivers, where the most urbanized sites showed higher MP contamination [25,26,36]. In the Costa-Couros river, although there were no significant differences among sites for the number of MPs in the sediments, the abundance of these particles increased along the river, with the lowest number of MPs occurring in the most upstream site (C1). However, the abundance of MPs in C1 was higher than those observed at all sites of Selho river, probably because it is located in a recreational area within the urban center and, therefore, has a strong influence of human activities. Our findings align with Nel et al. [30], who found higher abundances of MPs in the sediments of more densely populated sites and recreational areas.

Both rivers displayed a higher number of MPs with a size < 0.5 mm than MPs with a size > 0.5 mm at all sites, suggesting that the MPs contaminating both rivers are mainly primary MPs (such as microbeads that usually tend to range between >0.1 µm to ≤1 mm in size [61]). Primary MPs have been used in a variety of personal care products (e.g., toothpaste, shampoo, cosmetic products, shaving cream [13,62]), the plastic production industry (virgin pellets [23]), industrial cleaning products (e.g., scrubbers for removal of rust or paint), and air blasting technology [13,62,63]. For example, a great quantity of microbeads with an average particle size of 100 µm [64] or smaller [65] are used in cosmetic products. Furthermore, previous research also revealed that primary MPs are more common in urban freshwater systems than secondary MPs, with a dominance of microbeads in sediments [28,66].

Several physical factors (e.g., wind, salinity, temperature, precipitation, river current, and geomorphology) can influence the distribution of MPs in riverine sediments [57,62]. According to Hurley et al. [28], MPs are efficiently flushed from river catchments during flood events. Given that sediment samples of this study were collected during the summer, the high number of MPs found in both rivers can also be related to the lack of rain events and low river currents, very characteristic of this season, allowing the withholding of these particles in the sediment.

The Costa-Couros river showed a higher abundance of MPs in the sediments than the Selho river. As mentioned above, the Costa-Couros river crosses the city of Guimarães, which makes it more vulnerable to urbanization. Moreover, this river’s low current and flow characteristics, especially during summer, make it even more prone to MP contamination than most of the urban catchments of Guimarães, including the Selho river [28].

Oligochaeta are one of the most widely distributed freshwater taxa that ingest sediment, extracting nutrients mainly from the organic matter to feed themselves (limnivores) [67]. This way of feeding makes these organisms one of the most susceptible to the ingestion of MPs that tend to sink and accumulate in the sediments. Once ingested by benthic organisms, MPs have a very long residence period in their gut, which can be induced either by the particle’s aggregation within the organisms’ digestive tract or by the gut blockage with particles of similar size to their size-limited capacity of ingestion [68].

In Costa-Couros river, the most urbanized site (C3) stood out by showing a lower abundance of MPs in the digestive tract of Oligochaeta, while, in the Selho river, the most upstream site (S1), located in an agricultural and forestry area, was the one that presented the highest number of MPs inside the gut of Oligochaeta.

The lower number of MPs in Oligochaeta from C3, seems to be associated with the weight of the organisms (Table 5) since significant differences between sites were found for the weight of Oligochaeta, with higher weights occurring in C3 compared to the other sites. This might indicate that organisms from C1, C2, and C4 are suffering from malnutrition and digestive disorders, as ingestion of MPs reduces the absorption of nutrients [69]. The retention of MPs inside the gut might also lead to a false feeling of satiety and trigger an inflammation response with consequences in the organisms’ behavior and physiological status [68,70,71].

In Selho river, the high number of MPs inside the digestive tract of Oligochaeta does not seem to be associated with the low weight of the organisms. In this river, the organisms with higher weights had a higher abundance of MPs in their guts (Table 5), which seems to be in accordance with a study by Garcia et al. [26], which reported that the abundance of ingested MPs increased with organisms’ size. This can suggest that, contrary to what was observed in the Costa-Couros river, the quantity of MPs ingested by Oligochaeta was insufficient to cause obvious impairment in their development. Furthermore, according to the study of Rauchschwalbe et al. [72], the biomass and the abundance of meiobenthic organisms such as Oligochaeta, was not altered when exposed to MPs, whereas the biomass and abundance of other taxa was affected.

According to previous studies, a high number of low-density MPs can be found at the bottom of rivers due to the development of biofilms [13,62,73]. The development of these organic-rich aggregates can lead to a preferential ingestion of these MPs by limnivore organisms (such as *Tubifex* spp.; [69]). Rodriguez et al. [74] also reported that *Tubifex* spp. worms tend to prefer to ingest particles associated with organic material. The selectivity of Oligochaeta to ingest these aggregated particles can be the reason for the high levels of MPs found in the gut of organisms from the Costa-Couros and Selho rivers. However, further investigation is required to assess this hypothesis.

Some studies have already reported that MPs’ abundance inside sediment-dwelling organisms (*Chironomus* spp.) reflects the abundance in the sediment they inhabit [30,31]. However, our results do not seem to support these findings, since sampling sites with a higher number of MPs in sediments do not necessarily have higher number of MPs inside organisms’ guts, and the other way around. This is consistent with the findings of Garcia et al. [26], which observed that the ingestion of MPs was not correlated with environmental MP pollution.

Concerning the size of the MPs found within the digestive tract of the Oligochaeta from the Costa-Couros river, heavier organisms were capable of ingesting MPs with bigger sizes, while smaller particles seemed to be associated with organisms with lower weights (Table 4). That was evident in organisms from C3 and C4 sites, which presented the largest and smaller MP mean size, respectively. The reduction in and/or absence of the capacity of ingestion after MPs retention in the digestive tract depends on the number of particles needed to be ingested to achieve the maximal volumetric capacity of the digestive tract [68]. Accordingly, the greatest number of MPs found within the gut of the organisms in both rivers can result from the small size of the MP particles, which enabled an accumulation of these forms inside of the digestive tract until the maximal volumetric capacity was reached. In previous recent studies, a number of MPs (>500 items) ranging from 20 to 60 µm found in the gut of chironomids triggered an anti-inflammatory and immune response leading to an oxidative stress condition of larvae and an impairment of development and reproduction [31,68,75]. A similar condition could have happened to the Oligochaeta from Costa-Couros, especially the organisms collected at C1, C2 and C4, whose high ingestion of MPs led to a development shortage. Through the comparison of the mean size of the MPs extracted from the Oligochaeta’s gut with the dominance of the particles with size < 0.5 mm in the sediment of the sampling sites, it is evident that Oligochaeta ingested MPs within the size range responsible for the contamination of all sampling sites in both rivers studied. Moreover, the findings suggest that the ingestion of these MPs by aquatic organisms could be mainly unintentional [26].

When comparing the abundance of MPs in both rivers, the Costa-Couros river presented significantly more MPs inside the gut of Oligochaeta. This can highlight the fact that organisms from the Costa-Couros river could already be suffering alterations in their behavior and physiological status (e.g., lack of growth and development, oxidative stress, digestive orders) caused by the high levels of MPs ingested [31,32,68,75], contrary to organisms from Selho river that do not seem to be affected by these plastic particles [72]. As it happened in the sediments, in Oligochaeta, the number of MPs was also higher in the most urbanized river (Costa-Couros river), emphasizing that urbanization is a key driver of MP contamination in freshwater systems.

### 4.3. Potential Linkage between Ecological Status and Microplastic Abundance

Both C3 in Costa-Couros river and S3 in Selho river stood out from the other sites by showing a lower abundance of MPs in Oligochaeta, as well as lower IPtI_N_ value (C3 IPtI_N_: 0.27; S3 IPtI_N_: 0.36; C3 and S3 presented “poor” ecological status). High levels of nutrients and a macroinvertebrate community dominated by organic pollution-tolerant taxa such as Oligochaeta were also observed at these sites. Oligochaeta live buried in the substrate and swallow the sediments taking advantage of organic matter, and they may unintentionally ingest microplastics associated with it. Thus, the high abundance of Oligochaeta may help explain the lower abundance of MPs in the organisms collected at C3 and S3 (high number of Oligochaeta, less food available for each individual).

However, in Selho river, the sampling site with the highest abundance of MPs inside the gut of Oligochaeta (S1) also achieved a “good” ecological status based on the benthic macroinvertebrate community. Unlike C3 and S3, the S1 site showed a more diverse macroinvertebrate community with fewer organic pollution-tolerant organisms, including Oligochaeta. This may suggest a greater availability of food for Oligochaeta and, likewise, a higher availability of MPs, resulting in a higher ingestion of MPs per individual.

Overall, our results show that sites with at least a “good” ecological status do not necessarily have lower abundance of MPs in the sediments and organisms. In fact, MP contamination is pervasive across the sediments and Oligochaeta’s gut in both rivers, including at sites with “good” or “high” ecological status based on the biological quality elements benthic macroinvertebrates.

## 5. Conclusions

The ecological status assessment based on the benthic macroinvertebrate community showed that, in both rivers, the sampling sites that did not reach a “good” ecological status were those under higher urban pressure (S2, C2, and C3) or simultaneously under urban and agricultural pressure (S3 and C4). These pressures resulted in increased hydromorphological modifications affecting the benthic macroinvertebrate community. In general, the urban influence, especially if combined with the agricultural activity, was a key driver in the decrease in the ecological status of the rivers under study.

However, the ecological status of a water body is determined by the ecological quality element with the worst quality. Thus, due to the high concentration of nutrients (especially phosphorus) at all sites (and seasons) in both rivers (except C2), neither site achieved an overall “good” ecological status. In Costa-Couros river, the sites under higher urban pressure and simultaneously urban and agricultural pressure, stood out for presenting higher phosphorus concentrations (C3 and C4). In Selho river, the most downstream site (S4), despite being under agricultural influence, stood out from the others for showing lower phosphorus concentrations, which seems to be related to the existence of more naturalized banks. Therefore, rehabilitation of riparian strips and the creation of conditions to retain nutrients from agricultural and urban activities occurring in the areas surrounding the sampling sites, together with reduction of diffuse pollution along the rivers, are necessary to improve the ecological status of the Selho and Costa-Couros rivers.

The results also showed that MP contamination is widespread in the sediments and Oligochaeta from both rivers, and urbanization seems to be the main driver for MP contamination. In river Selho, the highest number of MPs in sediments was found in the site integrated in an urban area (S2), while, in Costa-Couros, which is an urban river crossing the city of Guimarães, the abundance of MPs in the sediments was similar in all sampling sites. A high number of MPs were also found inside the digestive tract of Oligochaeta. In Costa-Couros river, the high number of MPs seems to be associated with the low weight of the organisms, suggesting the existence of malnutrition and digestive disorders. On the contrary, the heaviest Oligochaeta in Selho river were those with the highest number of MPs.

This study also makes a first approach to a potential link between ecological status and the abundance of MPs in freshwater ecosystems. It showed that sites with higher ecological status do not necessarily have lower abundances of MPs. Therefore, since MPs have the potential to cause harm to ecosystems, organisms, and human health, it is essential to monitor not only the ecological status but also emerging micropollutants such as MPs in freshwater ecosystems to successfully implement mitigation and remediation strategies.

## Figures and Tables

**Table 1:** Sampling sites of the Selho (S1 to S4) and Costa-Couros (C1 to C4) rivers, their location (Parish or Union of Parishes in Guimarães municipality), and land use near river banks.

Site	Parish/Union of Parishes in the Left Bank (LB) and Right Bank (RB)	Coordinates	Land Use
S1	LB and RB: Parish of S. Torcato	41°29′11″ N, 8°15′16″ W	Forestry Agricultural Rural recreational area
S2	LB: Parish of Aldão RB: Union of Parishes of Selho S. Lourenço and Gominhães	41°27′52″ N, 8°16′51″ W	Artificial surfaces (housing)
S3	LB: Union of Parishes of Candoso S. Tiago and Mascotelos RB: Parish of Creixomil	41°26′14″ N, 8°19′21″ W	Agricultural Peri-urban recreational area
S4	LB: Parish of Serzedelo RB: Parish of Gondar	41°24′38″ N, 8°22′34″ W	Agricultural
C1	LB and RB: Parish of Costa	41°26′50″ N, 8°16′42″ W	Urban recreational area
C2	LB and RB: Union of Parishes of Oliveira, S. Paio and S. Sebastião	41°26′29″ N, 8°17′14″ W	Artificial surfaces (housing, commercial, transport, etc.) Urban recreational area
C3	LB: Parish of Creixomil RB: Parish of Urgezes	41°26′23″ N, 8°17′59″ W	Artificial surfaces (housing, commercial, transport, etc.)
C4	LB and RB: Parish of Creixomil	41°26′12″ N, 8°18′29″ W	Agricultural Peri-urban recreational area

**Table 2:** Spatial variation of the IPtI_N_ (North Invertebrate Portuguese Index) and the respective ecological quality class of the sampling sites of Selho (S1 to S4) and Costa-Couros (C1 to C4) rivers.

Sampling Site	IPtI_N_ (EQR)	Quality Class
S1	0.72	II
S2	0.44	III
S3	0.36	IV
S4	1.65	I
C1	0.79	II
C2	0.45	III
C3	0.27	IV
C4	0.40	IV

Note: IPtI_N_ ecological quality classes established for “small-sized stream of North of Portugal”: IPtI_N_ score ≥ 0.87 (class I—“high” ecological status); IPtI_N_ score 0.68–0.86 (class II—“good” ecological status); IPtI_N_ score 0.44–0.67 (class III—“moderate” ecological status); IPtI_N_ score 0.22–0.43 (class IV—“poor” ecological status).

**Table 3:** Spatial and temporal variation of the physicochemical parameters determined in Selho and Costa-Couros rivers, respectively: water temperature (Temp., °C), pH, dissolved oxygen concentration (DO, mg O_2_/L), percentage saturation of dissolved oxygen (%DO), conductivity (Cond., μS/cm), salinity (Sal., PSU), total dissolved solids (TDS, mg/L), nitrates (NO_3_^−^, mg/L), nitrites (NO_2_^−^, mg/L), ammonium ion (NH_4_^+^, mg/L), total phosphorus (P, mg/L), chemical oxygen demand (COD, mg/L), and biochemical oxygen demand (BOD_5_, mg/L). Data are presented as the mean ± SD.

		Sampling Site				Season/Year			
		S1	S2	S3	S4	Summer 2020	Autumn 2020	Winter 2021	Spring 2021
Selho river	Temp.	12.94 ± 1.62	14.46 ± 1.54	16.08 ± 2.21	14.74 ± 2.59	15.69 ± 1.81	12.34 ± 0.48	13.66 ± 1.05	16.53 ± 1.80
	pH	6.81 ± 0.53	6.71 ± 0.14	6.76 ± 0.22	6.99 ± 0.20	6.80 ± 0.36	7.05 ± 0.32	6.94 ± 0.06	6.51 ± 0.13
	DO	9.40 ± 1.62	9.14 ± 0.87	7.98 ± 1.37	9.16 ± 1.07	7.82 ± 1.42	8.81 ± 0.76	10.21 ± 0.94	8.84 ± 1.04
	%DO	92.18 ± 15.30	91.53 ± 7.78	84.37 ± 14.79	92.12 ± 7.92	81.05 ± 14.47	84.93 ± 6.27	100.20 ± 8.40	93.98 ± 7.56
	Cond.	51.50 ± 12.70	90.83 ± 23.96	141.8 ± 47.44	131.8 ± 39.6	136.50 ± 51.99	129.80 ± 49.84	70.75 ± 23.28	78.92 ± 25.58
	Sal.	0.03 ± 0.01	0.04 ± 0.01	0.07 ± 0.02	0.06 ± 0.02	0.07 ± 0.02	0.06 ± 0.02	0.03 ± 0.01	0.04 ± 0.01
	TDS	26.00 ± 6.89	45.25 ± 11.99	71.00 ± 23.56	65.75 ± 19.59	68.17 ± 25.73	65.08 ± 24.51	35.25 ± 11.97	39.50 ± 12.76
	NO_3_^−^	10.49 ± 2.71	20.96 ± 2.41	21.10 ± 4.60	22.05 ± 3.69	23.35 ± 5.74	17.13 ± 5.52	15.47 ± 4.39	18.65 ± 5.01
	NO_2_^−^	0.04 ± 0.01	0.05 ± 0.02	0.37 ± 0.31	0.18 ± 0.11	0.34 ± 0.10	0.17 ± 0.05	0.03 ± 0.01	0.05 ± 0.02
	NH_4_^+^	0.06 ± 0.08	0.10 ± 0.10	0.90 ± 0.63	0.12 ± 0.09	0.48 ± 0.73	0.45 ± 0.49	0.07 ± 0.06	0.18 ± 0.21
	P	0.38 ± 0.08	0.33 ± 0.09	0.28 ± 0.06	0.19 ± 0.03	0.30 ± 0.12	0.25 ± 0.07	0.33 ± 0.09	0.31 ± 0.11
	COD	10.50 ± 13.81	8.50 ± 11.52	9.33 ± 9.60	14.08 ± 12.11	5.17 ± 4.13	8.25 ± 4.77	0.00 ± 0.00	29.00 ± 3.52
	BOD_5_	3.47 ± 1.68	3.39 ± 0.77	2.75 ± 1.31	3.66 ± 1.15	2.58 ± 1.24	2.90 ± 0.66	4.90 ± 0.85	2.90 ± 0.75
		**C1**	**C2**	**C3**	**C4**	**Summer 2020**	**Autumn 2020**	**Winter 2021**	**Spring 2021**
Costa-Couros river	Temp.	14.72 ± 1.72	15.57 ± 2.35	15.96 ± 2.70	15.61 ± 1.86	18.52 ± 1.11	13.64 ± 0.22	13.54 ± 0.40	16.16 ± 0.57
	pH	6.99 ± 0.34	6.95 ± 0.21	7.07 ± 0.13	7.04 ± 0.03	7.04 ± 0.10	7.18 ± 0.18	7.06 ± 0.08	6.77 ± 0.20
	DO	7.89 ± 0.71	8.23 ± 1.32	7.98 ± 1.56	6.69 ± 0.78	8.57 ± 1.54	7.74 ± 0.90	8.25 ± 0.19	6.24 ± 0.36
	% DO	81.18 ± 4.51	85.40 ± 15.65	84.45 ± 18.66	69.88 ± 5.70	94.44 ± 18.62	76.97 ± 9.03	80.52 ± 1.53	68.99 ± 3.46
	Cond.	142.80 ± 43.12	151.50 ± 38.90	73.92 ± 64.41	215.80 ± 68.58	173.80 ± 102.60	159.00 ± 98.10	113.20 ± 20.56	138.00 ± 20.00
	Sal.	0.07 ± 0.02	0.07 ± 0.02	0.03 ± 0.03	0.10 ± 0.03	0.08 ± 0.05	0.08 ± 0.05	0.05 ± 0.01	0.06 ± 0.01
	TDS	71.25 ± 21.50	76.00 ± 19.50	37.00 ± 32.18	108.00 ± 34.15	89.11 ± 41.34	79.50 ± 49.06	56.58 ± 10.63	69.25 ± 9.75
	NO_3_^−^	19.07 ± 4.23	24.96 ± 5.38	18.16 ± 6.51	19.62 ± 12.67	29.72 ± 3.96	17.83 ± 5.12	12.52 ± 8.06	21.74 ± 1.35
	NO_2_^−^	0.16 ± 0.10	0.18 ± 0.07	0.30 ± 0.13	0.54 ± 0.21	0.48 ± 0.23	0.27 ± 0.17	0.15 ± 0.09	0.26 ± 0.16
	NH_4_^+^	0.17 ± 0.23	0.29 ± 0.20	1.32 ± 0.81	2.43 ± 2.07	1.73 ± 1.91	1.78 ± 1.66	0.31 ± 0.14	0.40 ± 0.34
	P	0.20 ± 0.07	0.10 ± 0.07	0.35 ± 0.13	0.34 ± 0.22	0.28 ± 0.20	0.25 ± 0.18	0.19 ± 0.16	0.28 ± 0.14
	COD	8.67 ± 3.89	2.25 ± 2.18	15.92 ± 4.06	12.50 ± 8.99	8.00 ± 5.77	9.17 ± 8.64	12.50 ± 6.25	9.65 ± 8.40
	BOD_5_	2.14 ± 1.09	3.00 ± 1.47	3.43 ± 1.69	1.82 ± 1.20	3.41 ± 1.84	2.36 ± 0.80	3.72 ± 0.04	0.88 ± 0.22

Note: Threshold values established for the “good” ecological status in Northern Portuguese rivers: pH (6–9); DO (≥5 mg O_2_/L); %DO (60–120%); NO_3_^−^ (≤25 mg/L); NH_4_^+^ (≤1 mg/L); P (≤0.10 mg/L); BOD_5_ (≤6 mg O_2_/L).

**Table 4:** Minimum, maximum, and mean ± SEM sizes of random samples of ten MPs existing in the digestive tract of Oligochaeta collected from four sampling sites of Selho (S1 to S4) and Costa-Couros (C1 to C4) rivers.

	Selho River				Costa-Couros River			
	S1	S2	S3	S4	C1	C2	C3	C4
Min. (μm)	20	20	10	20	20	30	40	20
Max. (μm)	60	70	70	60	70	70	90	40
Mean (μm)	42	35	34	36	44	47	53	31
±SEM	±5.12	±6.01	±6.18	±4.99	±5.62	±3.96	±4.96	±3.15

**Table 5:** Minimum, maximum, and mean ± SEM weight of the Oligochaeta collected from Selho (S1 to S4) and Costa-Couros (C1 to C4) rivers, analyzed after removing outliers.

	Selho River				Costa-Couros River			
	S1	S2	S3	S4	C1	C2	C3	C4
Min. (g)	0.008	0.007	0.006	0.008	0.076	0.007	0.158	0.008
Max. (g)	0.169	0.021	0.017	0.215	0.134	0.520	0.744	0.019
Mean (g)	0.080	0.014	0.011	0.063	0.111	0.081	0.464	0.010
±SEM	±0.013	±0.001	±0.001	±0.015	±0.005	±0.046	±0.045	±0.001

## Data Availability

Not applicable.

## Supplementary Materials

The following supporting information can be downloaded at https://www.mdpi.com/article/10.3390/ijerph192315956/s1: Figure S1. Spatial and seasonal variation of the physicochemical parameters determined in Selho river; Figure S2. Spatial and seasonal variation of the physicochemical parameters determined in Costa-Couros river; Table S1. Two-way ANOVA applied to the physicochemical parameters of Selho and Costa-Couros rivers.
