# Supplementary material for: Microplastic Contamination and Ecological Status of Freshwater Ecosystems: A Case Study in Two Northern Portuguese Rivers
Source: Int J Environ Res Public Health. 2022 Nov 30;19(23):15956. doi: 10.3390/ijerph192315956 (PMC9735593; doi:10.3390/ijerph192315956)
Supplement: Supplementary file 1 [file ijerph-19-15956-s001.zip › ijerph-2015468-supplementary.pdf]

International Journal of
Environmental Research and Public Health

MDPI

Spatial and seasonal variation of the physicochemical parameters determined in Selho river

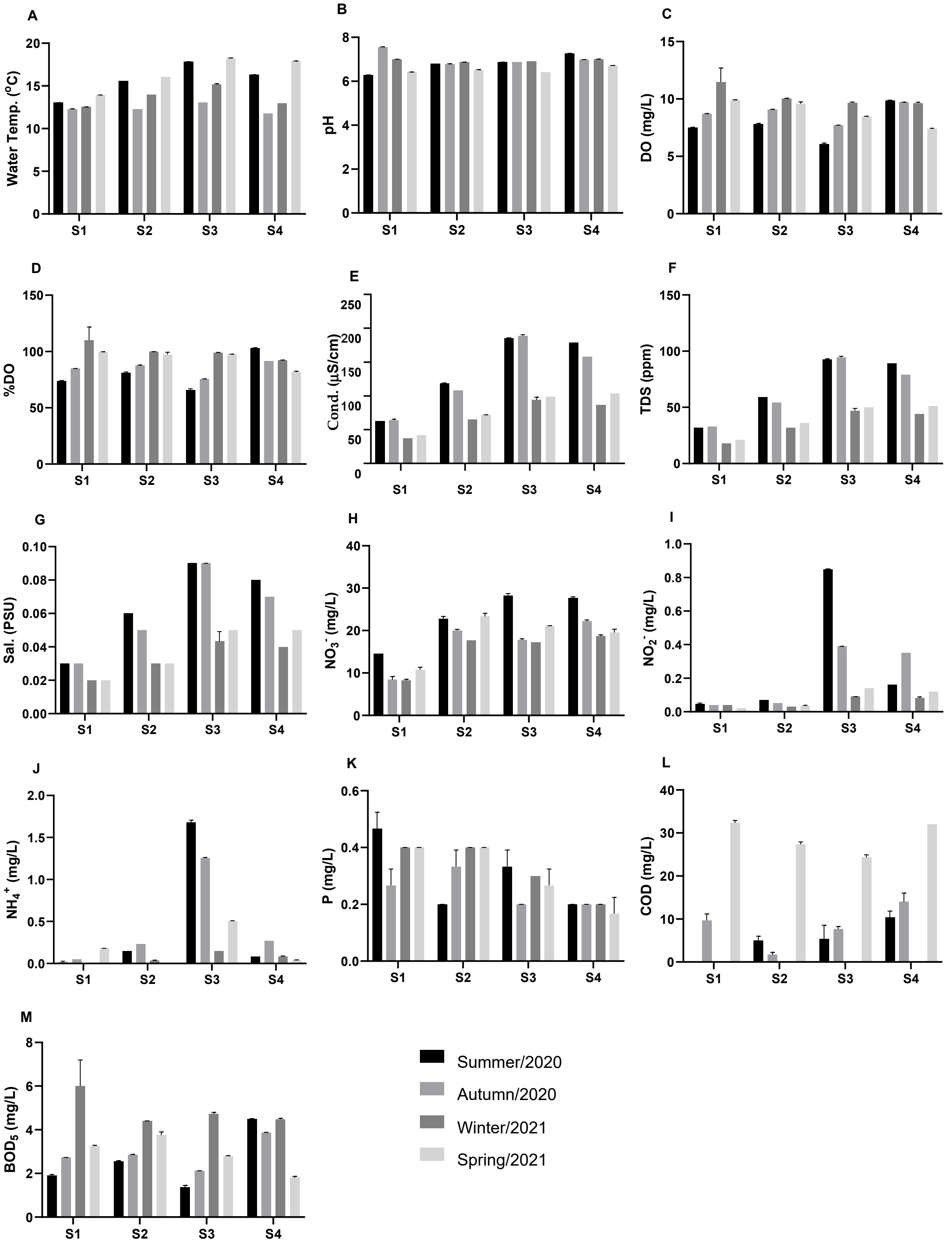

Figure S1. Spatial and seasonal variation of the physicochemical parameters determined at the sampling sites of Selho river (S1 to S4) in different seasons (summer and autumn of 2020, winter and spring of 2021). **A** Water temperature, Water Temp.; **B** pH; **C**

Dissolved oxygen concentration, DO; **D** Percent saturation of dissolved oxygen, %DO; **E** Conductivity, Cond.; **F** Salinity, Sal.; **G** Total dissolved solids, TDS; **H** nitrates, NO_3^-; **I** Nitrites, NO_2^-; **J** Ammonium ion, NH_4^+; **K** Total phosphorus, P; **L** Chemical oxygen demand, COD; **M** Biochemical oxygen demand, BOD_5. Data are presented as mean ± standard deviation.

Spatial and seasonal variation of the physicochemical parameters determined in Costa-Couros river

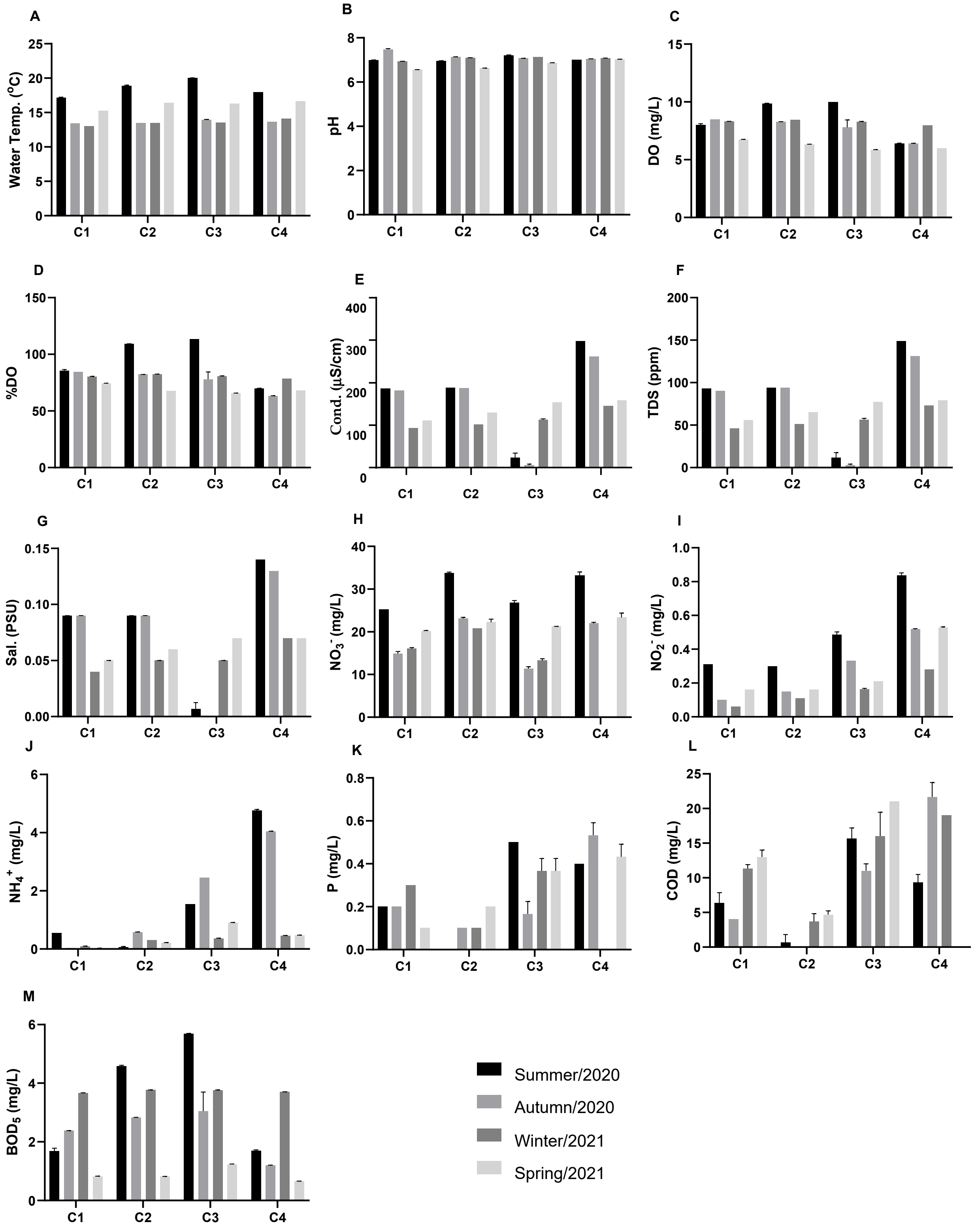

Figure S2. Spatial and seasonal variation of the physicochemical parameters determined at the sampling sites of Costa-Couros river (C1 to C4) in different seasons (summer and autumn of 2020, winter and spring of 2021). **A** Water temperature, Water Temp.; **B** pH; **C** Dissolved oxygen concentration, DO; **D** Percent saturation of dissolved oxygen, %DO; **E** Conductivity, Cond.; **F** Salinity, Sal.; **G** Total dissolved solids, TDS; **H** nitrates, NO_3^-; **I** Nitrites, NO_2^-; **J** Ammonium ion, NH_4^+; **K** Total phosphorus, P; **L** Chemical oxygen demand, COD; **M** Biochemical oxygen demand, BOD_5. Data are presented as mean ± standard deviation.

Two-way ANOVA applied to the physicochemical parameters

Table S1. Results from the two-way ANOVA applied to the physicochemical parameters determined in Selho and Costa-Couros rivers: water temperature (Temp., °C), pH, dissolved oxygen concentration (DO, mg O_2/L), percent saturation of dissolved oxygen (%DO), conductivity (Cond., μS/cm), salinity (Sal., PSU), total dissolved solids (TDS, mg/L), nitrates (NO_3^-, mg/L), nitrites (NO_2^-, mg/L), ammonium ion (NH_4^+, mg/L), total phosphorus (P, mg/L), chemical oxygen demand (COD, mg/L) and biochemical oxygen demand (BOD_5, mg/L). A significance level of 0.05 was considered for all tests.

		Spatial differences		Seasonal differences		Interaction	
		% of total variation	ANOVA Summary	% of total variation	ANOVA Summary	% of total variation	ANOVA Summary
Selho river	Temp.	27,37%	F (3, 32) = 21945<br>P<0,0001	60,17%	F (3, 32) = 48247<br>P<0,0001	12,45%	F (9, 32) = 3327<br>P<0,0001
	pH	9,48%	F (3, 32) = 2050<br>P<0,0001	40,72%	F (3, 32) = 8804<br>P<0,0001	49,75%	F (9, 32) = 3585<br>P<0,0001
	DO	17,23%	F (3, 32) = 51,91<br>P<0,0001	40,93%	F (3, 32) = 123,3<br>P<0,0001	38,30%	F (9, 32) = 38,46<br>P<0,0001
	%DO	7,58%	F (3, 32) = 19,77<br>P<0,0001	39,62%	F (3, 32) = 103,3<br>P<0,0001	48,71%	F (9, 32) = 42,32<br>P<0,0001
	Cond.	55,14%	F (3, 32) = 14278<br>P<0,0001	37,12%	F (3, 32) = 9611<br>P<0,0001	7,70%	F (9, 32) = 664,9<br>P<0,0001
	Sal.	54,63%	F (3, 32) = 2137<br>P<0,0001	37,04%	F (3, 32) = 1449<br>P<0,0001	8,05%	F (9, 32) = 105,0<br>P<0,0001
	TDS	55,12%	F (3, 32) = 14362<br>P<0,0001	37,68%	F (3, 32) = 9819<br>P<0,0001	7,16%	F (9, 32) = 622,1<br>P<0,0001
	NO_3^-	67,06%	F (3, 32) = 1875<br>P<0,0001	25,92%	F (3, 32) = 724,7<br>P<0,0001	6,64%	F (9, 32) = 61,86<br>P<0,0001
	NO_2^-	41,45%	F (3, 32) = 34202<br>P<0,0001	19,38%	F (3, 32) = 15994<br>P<0,0001	39,15%	F (9, 32) = 10769<br>P<0,0001
	NH_4^+	55,77%	F (3, 32) = 26687<br>P<0,0001	14,23%	F (3, 32) = 6809<br>P<0,0001	29,97%	F (9, 32) = 4780<br>P<0,0001
	P	53,18%	F (3, 32) = 65,11<br>P<0,0001	8,17%	F (3, 32) = 10,00<br>P<0,0001	29,95%	F (9, 32) = 12,22<br>P<0,0001
	COD	3,40%	F (3, 32) = 53,64<br>P<0,0001	91,04%	F (3, 32) = 1435<br>P<0,0001	4,88%	F (9, 32) = 25,66<br>P<0,0001
	BOD_5	7,39%	F (3, 32) = 20,24<br>P<0,0001	53,46%	F (3, 32) = 146,3<br>P<0,0001	35,25%	F (9, 32) = 32,16<br>P<0,0001
Costa-Couros	Temp.	4,51%	F (3, 32) = 2408<br>P<0,0001	91,23%	F (3, 32) = 48706<br>P<0,0001	4,24%	F (9, 32) = 755,3<br>P<0,0001

river	pH	4,53%	F (3, 32) = 217,4 P<0,0001	52,10%	F (3, 32) = 2502 P<0,0001	43,15%	F (9, 32) = 690,6 P<0,0001
	DO	22,61%	F (3, 32) = 207,7 P<0,0001	51,12%	F (3, 32) = 469,5 P<0,0001	25,10%	F (9, 32) = 76,84 P<0,0001
	% DO	20,48%	F (3, 32) = 221,5 P<0,0001	45,52%	F (3, 32) = 492,5 P<0,0001	33,02%	F (9, 32) = 119,1 P<0,0001
	Cond.	47,41%	F (3, 32) = 4640 P<0,0001	9,77%	F (3, 32) = 955,9 P<0,0001	42,71%	F (9, 32) = 1394 P<0,0001
	Sal.	47,73%	F (3, 32) = 4857 P<0,0001	10,31%	F (3, 32) = 1049 P<0,0001	41,85%	F (9, 32) = 1420 P<0,0001
	TDS	47,58%	F (3, 32) = 4304 P<0,0001	9,76%	F (3, 32) = 882,7 P<0,0001	42,54%	F (9, 32) = 1283 P<0,0001
	NO_3^-	10,95%	F (3, 32) = 491,8 P<0,0001	61,23%	F (3, 32) = 2749 P<0,0001	27,58%	F (9, 32) = 412,8 P<0,0001
	NO_2^-	57,19%	F (3, 32) = 10408 P<0,0001	35,32%	F (3, 32) = 6428 P<0,0001	7,42%	F (9, 32) = 450,2 P<0,0001
	NH_4^+	41,81%	F (3, 32) = 95329 P<0,0001	24,68%	F (3, 32) = 56263 P<0,0001	33,51%	F (9, 32) = 25471 P<0,0001
	P	38,86%	F (3, 32) = 166,6 P<0,0001	4,15%	F (3, 32) = 17,80 P<0,0001	54,50%	F (9, 32) = 77,89 P<0,0001
	COD	48,95%	F (3, 32) = 238,2 P<0,0001	5,20%	F (3, 32) = 25,32 P<0,0001	43,66%	F (9, 32) = 70,82 P<0,0001
	BOD_5	19,30%	F (3, 32) = 245,5 P<0,0001	56,88%	F (3, 32) = 723,3 P<0,0001	22,98%	F (9, 32) = 97,39 P<0,0001
